# Using Clinical Data Visualizations in Electronic Health Record User Interfaces to Enhance Medical Student Diagnostic Reasoning: Randomized Experiment

**DOI:** 10.2196/38941

**Published:** 2023-04-13

**Authors:** Lucille Cheng, Yalini Senathirajah

**Affiliations:** 1 School of Medicine University of Pittsburgh Pittsburgh, PA United States; 2 Department of Biomedical Informatics University of Pittsburgh Pittsburgh, PA United States

**Keywords:** electronic health record, EHR, System-1–type diagnostic reasoning, type-1 reasoning, diagnostic, diagnosis, user interface, user design, heuristics, medical education, clinical reasoning, reasoning process, data visualization, hGraph, cognitive burden, cognitive load, medical student, medical school

## Abstract

**Background:**

In medicine, the clinical decision-making process can be described using the dual-process theory consisting of the fast, intuitive “System 1,” commonly seen in seasoned physicians, and the slow, deliberative “System 2,” associated with medical students. System-1—type diagnostic reasoning is thought to be less cognitively burdensome, thereby reducing physician error. To date, limited literature exists on inducing System-1–type diagnosis in medical students through cognitive heuristics, particularly while using modern electronic health record (EHR) interfaces.

**Objective:**

In this experimental pilot study, we aimed to (1) attempt to induce System-1—type diagnostic reasoning in inexperienced medical students through the acquisition of cognitive user interface heuristics and (2) understand the impact of clinical patient data visualizations on students' cognitive load and medical education.

**Methods:**

The participants were third- and fourth-year medical students recruited from the University of Pittsburgh School of Medicine who had completed 1+ clinical rotations. The students were presented 8 patient cases on a novel EHR, featuring a prominent data visualization designed to foster at-a-glance rapid case assessment, and asked to diagnose the patient. Half of the participants were shown 4 of the 8 cases repeatedly, up to 4 times with 30 seconds per case (Group A), and the other half of the participants were shown cases twice with 2 minutes per case (Group B). All participants were then asked to provide full diagnoses of all 8 cases. Finally, the participants were asked to evaluate and elaborate on their experience with the system; content analysis was subsequently performed on these user experience interviews.

**Results:**

A total of 15 students participated. The participants in Group A scored slightly higher on average than those in Group B, with a mean percentage correct of 76% (95% CI 0.68-0.84) versus 69% (95% CI 0.58-0.80), and spent on average 50% less time per question than Group B diagnosing patients (13.98 seconds vs 19.13 seconds, *P*=.03, respectively). When comparing the novel EHR design to previously used EHRs, 73% (n=11) of participants rated the new version on par or higher (3+/5). Ease of use and intuitiveness of this new system rated similarly high (mean score 3.73/5 and 4.2/5, respectively). In qualitative thematic analysis of poststudy interviews, most participants (n=11, 73%) spoke to “pattern-recognition” cognitive heuristic strategies consistent with System 1 decision-making.

**Conclusions:**

These results support the possibility of inducing type-1 diagnostics in learners and the potential for data visualization and user design heuristics to reduce cognitive burden in clinical settings. Clinical data presentation in the diagnostic reasoning process is ripe for innovation, and further research is needed to explore the benefit of using such visualizations in medical education.

## Introduction

In medicine, the clinical decision-making process can be described using the dual-process theory, which postulates cognitive processes consist of the fast, intuitive System 1 and the slow, deliberative System 2 [1-5]. Colloquially, System 1 is described as “your gut feeling” and requires minimal cognitive effort due to the use of past experiences and heuristics, whereas System 2 requires significant cognitive effort and can be associated with hypothesis creation and testing [6]. The System-1–type diagnostic reasoning is assumed to take years of experience to develop and is commonly seen in seasoned physicians, whereas the System-2–type diagnostic reasoning is more associated with learners, such as medical students [7].

System-1–type diagnostic reasoning in novices may be possible through the acquisition of cognitive heuristics. One example is Rosby et al [8], who accomplished this by training students to use System-1–type diagnostic reasoning via rapid repeated exposures to training x-rays and showing that this was effective in contrast to longer, fewer exposures. The less cognitively burdensome System-1–type diagnostic reasoning has benefits for patients by allowing physicians to be more present and reducing the potential for mistakes. Human cognitive capacity is limited and prone to error when overtaxed, yet health care systems require physicians to complete efficiently and accurately several, often unrelated, tasks simultaneously [9-11]. Newly developed tools which can provide a “snapshot” of relevant information and live alongside “visualization tools and graphical representations that better synthesize patient information” have been cited as promising approaches moving forward with electronic health records (EHRs) [9,11].

In this experimental study, we aim to further explore the work done by Rosby et al [8] and the potential of data visualizations in EHRs in the two following ways: (1) to induce System-1–type diagnostic reasoning in inexperienced medical students through the acquisition of cognitive user interface heuristics and (2) to better understand the impact of clinical patient data visualizations on students' cognitive load and medical education.

## Methods

### Ethical Considerations

This study received approval from the institutional review board of the University of Pittsburgh Human Research Protection Office under STUDY19020169.

### Statistical Analysis

Under institutional review board approval from the University of Pittsburgh, the participants recruited were 15 third- and fourth-year medical students who had completed at least one clinical rotation (Table 1). Students were first asked about their experience with existing EHR products, and basic demographic information was collected. They were then randomly assigned to 1 of 2 groups, Group A or Group B. Similarity between the 2 groups was assessed with the Welch 2-tailed *t* test and ANOVA of the demographic information collected (Table 1). Subsequently, the participants underwent the 3 steps of the study, which were familiarization, training, and testing.

**Figure 1:**
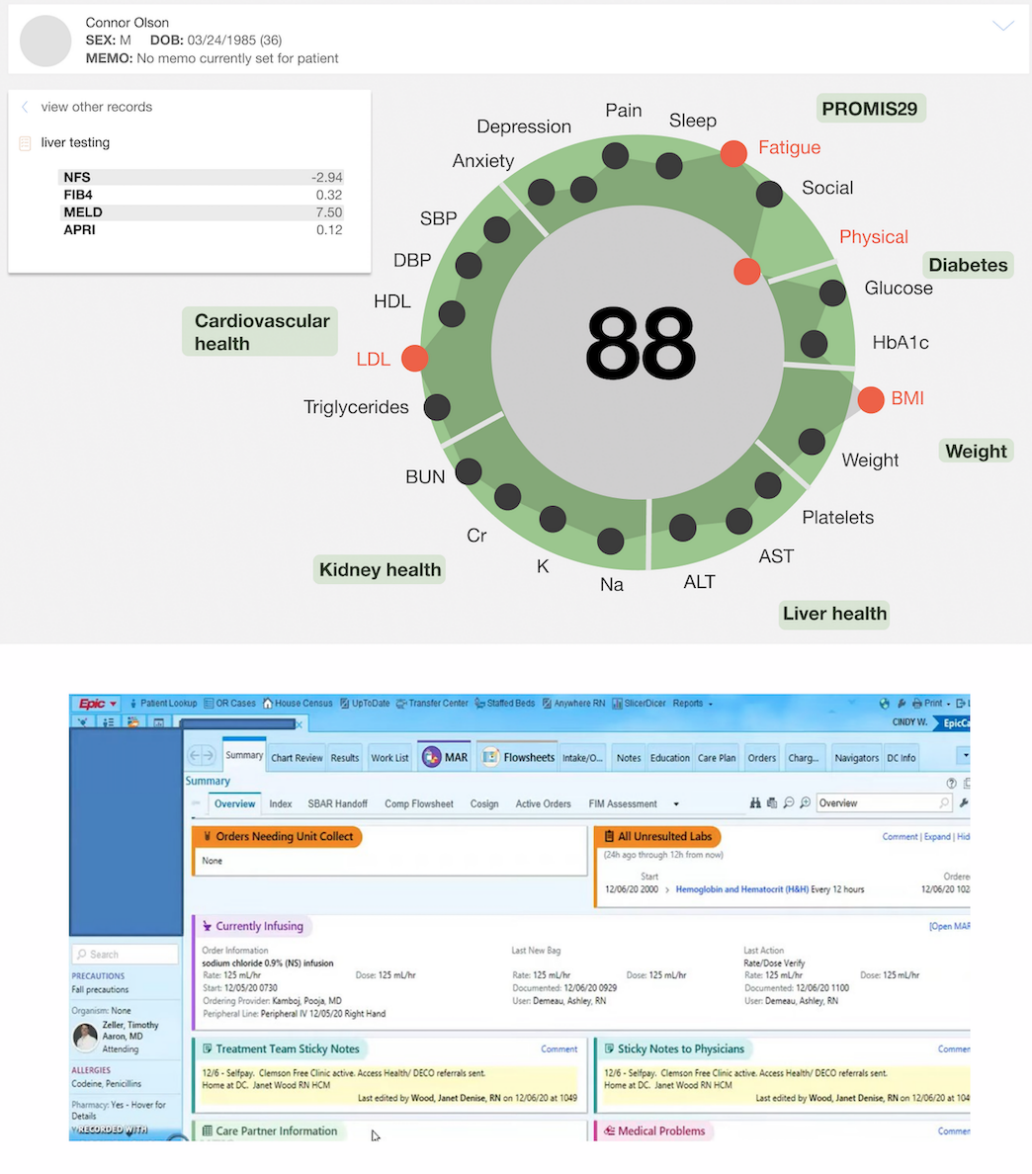
One of 8 cases participants were familiarized with, then later asked to diagnose (top) compared to the conventional electronic health record (EHR) screen from Epic all students reported previously using (bottom, via emrsystems.net). All cases were displayed on the same user interface which is based on the open-source visualization hGraph, (hgraph.org). The green circle represents the normal range for the parameters shown. The gray "shadow" formed by the linkage of all the values is intended to allow the user to see patterns that may help in future pattern recognition. ALT: alanine transaminase; APRI: AST to platelet ratio index; AST: aspartate aminotransferase; BUN: blood urea nitrogen; Cr: creatinine; DOB: date of birth; DBP: diastolic blood pressure; FIB4: fibrosis-4; HbA_1c_: hemoglobin A_1c_; HDL: high-density lipoprotein; K: potassium; LDL: low-density lipoprotein; MELD: model for end-stage liver disease; Na: sodium; NFS: nonalcoholic fatty liver disease fibrosis score; PROMIS29: patient-reported outcomes measurement information system; SBP: systolic blood pressure.

In the familiarization phase, all participants were shown 8 cases based on real patients with clinical information indicative of nonalcoholic fatty liver disease (NAFLD) or metabolic syndrome. The correct diagnosis of each of the patients was one of the following: (1) has metabolic syndrome and NAFLD, (2) has metabolic syndrome and does not have NAFLD, (3) does not have metabolic syndrome and has NAFLD, or (4) does not have metabolic syndrome nor NAFLD. The correct diagnosis for each case was given to the participants, and case information was displayed on a novel EHR user interface featuring a prominent data visualization component (Figure 1).

**Figure 2:**
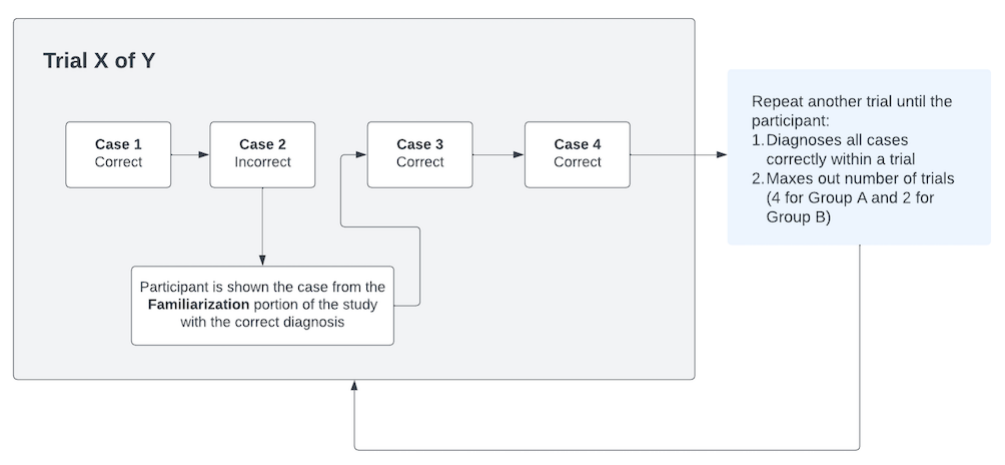
A schematic example of a trial, consisting of 4 cases. In this example, a student correctly diagnosed Case 1, then incorrectly diagnosed Case 2. They were subsequently shown the correct diagnosis and case slide from the familiarization portion of the study. After seeing the correct answer, they went on to diagnose Case 3 and Case 4 correctly. However, since they misdiagnosed 1 of the 4 cases, they needed to undergo another trial (ie, repeat all 4 cases) until they could either diagnose all cases correctly within 1 trial or max out the number of trials for their assigned group.

Next, students in each group were shown 4 cases and asked to provide a correct, full diagnosis (ie, “has metabolic syndrome and NAFLD”) for all 4 cases in a row or 1 trial (Figure 2). Students in Group A were shown each case within a trial up to 30 seconds per case for a total of 4 trials maximum, whereas Group B participants were shown each case within a trial for up to 2 minutes per case for a total of 2 trials maximum. If the students did not correctly diagnose all 4 cases within a trial before maxing out their allotted trial repeats, they would automatically be moved to the testing phase. During the final test phase, all participants were shown all 8 patient cases and asked to provide a full diagnosis of the patient. There was no time limit for either group.

After the study, the participants were asked to evaluate and elaborate on their experience with the novel EHR design. The questions asked included the following: (1) rating ease of system, (2) rating intuitiveness of system, (3) rating usefulness of system, (4) comparing the novel system with past EHRs used based on intuitiveness, (5) strategies used to compete the tasks, and (6) missing features that would have helped the completion of task and areas for improvement. Questions that asked the participants to give a rating or comparison were formatted on a scale of 1-5, where 1 was the low end of the spectrum (ie, very difficult if asking to rate ease of the system) and 5 being the high end of the spectrum (ie, very easy in the aforementioned example). The other questions were open-ended, and the study facilitators encouraged the participants to speak freely in this section. Unbiased follow-up questions eliciting clarification from the participants were occasionally asked. The Welch *t* test was performed on quantitative values using STATA/SE (StataCorp) and qualitative thematic analysis using the MAXQDA VERBI software was performed on this user interview portion of the study.

**Table 1:** Summary table of participant demographics.

Characteristics			Group A (n=8), n (%)		Group B (n=7), n (%)	*P* value	
**Age (years)**							.08
	<25	2 (25)		0 (0)			
	>25	6 (75)		7 (100)			
**Sex**							.43
	Male	4 (50)		5 (71)			
	Female	4 (50)		2 (29)			
**Class year**							.74
	MS3^a^	3 (38)		2 (29)			
	MS4	4 (50)		4 (57)			
	Other	1 (13)		1 (14)			
**Time (hours/week) spent browsing the internet**							.98
	>16	3 (38)		3 (43)			
	11-15	2 (25)		2 (29)			
	<10	3 (38)		2 (29)			
**EHR^b^ usage frequency (days/week)**							.42
	≥5	5 (63)		6 (86)			
	2-4	1 (13)		1 (14)			
	<1	2 (25)		0 (0)			
**EHR products used**							
	Cerner	8 (100)		7 (100)		—^c^	
	Epic	8 (100)		7 (100)		—	
	Other^d^	3 (38)		4 (57)		.23	

^a^MS: medical student.

^b^EHR: electronic health record.

^c^Not applicable.

^d^Centricity, Aria, and Computerized Patient Record System (CPRS).

## Results

A total of 15 medical students participated in the study. Participants in Group A scored slightly higher on average than participants in Group B, with a mean percentage correct of 76% (95% CI 0.68-0.84) versus 69% (95% CI 0.58-0.80) during the final testing portion. While we fail to reject the null hypothesis (*P*=.40), the participants in Group A spent, on average, 50% less time per question than Group B diagnosing patients during the final, time-unlimited testing portion (13.98 seconds vs 19.13 seconds, *P*=.03). A 2-sample equal variance (independent) 2-tailed *t* test was performed. The difference was found to be significant but inconclusive due to the small sample size (Table 2).

All participants in both groups had previously used Epic and Cerner, and none of the participants in either groups differed significantly in their perceptions of ease of use or usefulness of these EHRs (Table 3). When comparing the study EHR design to previously used EHRs, both groups on average rated the study EHR on par or higher than the existing EHRs (mean score 3.38/5.0 vs 3.71/5.0 for Group A vs B, respectively). Moreover, 73% (n=11) of all participants rated the new version on par or higher than existing EHRs; the ease of use and intuitiveness of this new system rated similarly high.

Qualitative thematic analysis revealed participants across both groups spoke positively about the visual representation data, in particular the ease in quickly assessing a patient’s overview (n=11, 73.3%), the consistency of the user interface layout and reducing number of clicks (n=10, 66.7%), and intuitive color coding (n=8, 53.3%; Table 4). When asked “What strategies did you utilize to help you complete this task?” some participants discussed pattern recognition (n=8, 53.3%) or using the consistency of the user interface (n=10, 66.7%) in combination with their prior clinical training. Areas of improvement for the interface were including numerical values for patient labs to gauge the severity of the condition (n=12, 80.0%) and more clarification around the central “health score” of the patient (n=7, 46.7%).

**Table 2:** Key analysis of the testing portion of the experiment, split by groups. Major results of interest included the accuracy of diagnoses (represented by mean % correct) and the speed of diagnosis (represented by mean time spent per question).

Testing	Group A (n=8)	Group B (n=7)	*P* value
Percentage of correct questions (95% CI)	76 (68-84)	69 (58-80)	.40
Seconds spent per question	13.98	19.13	.03

**Table 3:** Interview questions asked and corresponding responses. All questions were “on a scale of 1-5,” where 1 is the low end of the spectrum (ie, very difficult) and 5 is the high end of the spectrum (ie, very easy) for questions pertaining to ease of use.

Variables		Values, mean (SD)					
		Group A (n=8)		Group B (n=7)		*P* value	
**Perceived ease of use**							
	Epic	3.38 (0.92)	3.43 (0.53)		.89		
	Cerner	2.88 (0.99)	2.43 (0.53)		.29		
	Study EHR^a^	3.63 (0.92)	3.86 (1.07)		.66		
**Perceived usefulness**							
	Epic	4.38 (0.74)	4.29 (0.76)		.82		
	Cerner	4.25 (0.89)	3.86 (0.90)		.41		
	Study EHR	3.75 (0.71)	3.57 (0.79)		.65		
**Perceived intuitiveness**							
	Study EHR	4.13 (0.83)	4.29 (1.11)		.76		
Ease of use comparison of study EHR to familiar EHR		3.38 (1.30)		3.71 (1.11)		.59	

^a^EHR: electronic health record.

**Table 4:** Key themes and choice quotes from the participants based on MAXQDA analysis.

Theme	Frequency	Quote
Ability to quickly assess a patient’s overall health	11	“I really like the wheel concept because you’re getting a picture of every component of the patient’s health” (Group A participant)
Consistency of interface layout and reduced number of clicks aiding ease of use	10	“This was a lot more intuitive than [other EHR^a^] where it’s just a bunch of abnormal labs you have to double click to see if it’s high or low” (Group B participant)
Intuitive color-coding aiding ease of use	8	“Once I got used to it…visually, it was very easy to see that a bright orange cluster was a [metabolic syndrome] cluster” (Group B participant)
Pattern recognition as a strategy used to accomplish task	8	“It felt very natural to look at the right areas…after a few patients, my eyes were moving where they needed to go” (Group A participant)
Desire to have numerical lab values included to gauge the severity of the patient’s condition	12	“It was nice to see trends over times, but without a number I don’t know what the patient’s baseline is…” (Group A participant)
Confusion around central “health score” (ie, large number in the middle of the data visualization)	7	“I knew [the number] was important, but I didn’t know what information it was conveying” (Group B participant)

^a^EHR: electronic health record.

## Discussion

### Principal Results

In this study, we attempted to induce System-1 diagnostic reasoning in medical students by using a novel EHR data visualization design. Despite the failure to reject the null hypothesis, we observed a statistically significant difference in the amount of time Group A participants took to fully diagnose patient cases compared with Group B. The increased speed of diagnosis is a key component in System-1–type diagnostic reasoning, as physicians are presumed to rely on pattern recognition based on their past experiences and heuristics as opposed to exerting cognitive effort on the spot. This finding, coupled with the trending results of more accurate diagnoses by Group A than Group B, is suggestive of the ability to induce an accurate System-1–type clinical diagnostic reasoning ability in medical students using frequent repeat exposures. This is akin to the findings by Rosby et al [8].

For students to successfully accomplish the given task of diagnosing whether a patient had NFLD or metabolic syndrome all in a few minutes, several spoke about using “pattern-recognition” cognitive heuristic strategies consistent with System 1 decision-making. These patterns generally fell into one of the following three categories: (1) consistency of layout aiding in finding specific lab values, (2) trends between different lab values and subsequent diagnosis, and (3) visualization-specific features such as color coordination. One participant spoke of the “search pattern” they had developed through medical school and believed was represented in the layout of the user interface, stating the following:

the way the page was set up, it felt very natural to look at the right areas. I would look at BMI first, then down at ALT and AST [common lab values for diagnosing metabolic syndrome and non-alcoholic fatty liver disease]…after a few patients, my eyes were moving where they needed to go.

### Limitations

Our study had several limitations. First, it was only performed with 15 students at 1 academic institution, thereby making generalizability unlikely. Additionally, only 1 data visualization interface was shown to all participants. The specific design that was used may not adequately represent other potential iterations of clinical data visualizations on EHRs and again makes generalizability unlikely.

We also did not compare the efficacy of the novel EHR with an existing EHR interface such as Epic or Cerner, as our participant criteria included previous EHR experience, and we were interested in the ability to induce System-1 thinking with a completely novel system. We chose to limit our study to participants who had previously used some sort of EHR as these participants were able to provide us design feedback informed by their past clinical experience, as opposed to purely aesthetic feedback on the novel EHR design.

Finally, the incorporation of data visualization into EHRs is limited to the decision of the EHR companies; while there may be some benefit to teaching students clinical data through more illustrative methods, this benefit may be moot if visualizations are not adopted on the primary platform where students perform their clinical duties.

### Further Considerations

Several of the qualitative themes hold promise in further investigation of amalgamating the current offerings of how medical education is delivered. Many of the issues students mentioned with current EHRs are solved usability problems in the consumer technology industry by companies such as Google and Apple, but the solutions are not widely adopted in health care today [12,13]. Similarity, the value of data visualizations is not new [14-16], but to our knowledge, this type of clinician-side data visualization is not widely used in medical education.

The heuristics participants alluded to mirror the widely accepted “10 Usability Heuristics” in consumer user experience web design by Nielsen [17], or foundational principles established by Nielsen in 1994 for evaluating the usability of website interfaces [18]. We will focus on the following 2 in particular: Heuristic #4, or “Consistency and Standards,” as well as Heuristic #8, “Aesthetic and Minimalist Design.”

We begin first by talking about Heuristic #4, which states that “users should not have to wonder whether different words, situations or actions mean the same thing. Follow platform and industry conventions” [19]. When taken into consideration with the Jakob Law, or the fact that most users are spending their time on products *other* than EHRs; introducing a new interface through an EHR that works differently from the consumer products users are accustomed to increases cognitive load by forcing them to learn something new [20]. Data visualizations and illustrative representations of data have become increasingly common user interfaces in consumer technology products such as Jawbone UP and Fitbit [21]. The efficacy of modeling the novel EHR interface after these known patterns was reflected in the higher-than-average intuitiveness scores given by most participants. One noted that the interface “looks like something you’d show a patient…like it would be on the front page of [the patient facing hospital account portal]*.*” This serves as a good reminder that medical students, in addition to becoming physicians, are patients and technology consumers who have to context switch every time they use present-day EHRs.

Heuristic #8, Aesthetic and Minimalist Design, builds upon the basis set by Heuristic #4 and states, “interfaces should not contain information which is irrelevant or rarely needed. Every extra unit of information in an interface competes with the relevant units of information and diminishes their relative visibility” [19]. The novel EHR interface shown to participants was built on hGraph, whose creators were inspired to reduce problems in the health care experience resulting from an excess of data. They did so by using the “single picture method,” which compiled multiple metrics into a unified graph with the belief that “healthcare information visualizations should enable pattern recognition” [21]. In our study, participants spoke to this inadvertently through their comments about the ability to quickly assess a patient’s overall health. In total, 73% of the participants appreciated the ability to easily see an overview of their patients’ health and intuited that they would be able to get more details in a more interactive version of the interface. These heuristics are especially important considering the influence technology has had on our participants’, and broadly, current millennial medical students’ visual consumption of content [22].

### Conclusions

How clinical data are presented in the diagnostic reasoning process and medical education is ripe for innovation. In this study, students were able to diagnose patients more accurately after short, repeated exposure to the data visualization interface, implying the possibility of inducing type-1 diagnostics. Additionally, this study demonstrates how incorporating data visualizations and user design heuristics during care delivery can potentially reduce cognitive burden and allow even novices to diagnose quickly and correctly. Further experiments on different, visual displays of data and the benefits it may have on medical education should be conducted, especially in comparison to the existing commonly used EHRs. Studies using eye tracking to better understand what patterns students used, as well as which features were most or least used, should also be run to more precisely understand the search patterns mentioned by the students.

## Abbreviations

EHR: electronic health record
NAFLD: nonalcoholic fatty liver disease
